# Development, evaluation and application of 3D QSAR Pharmacophore model in the discovery of potential human renin inhibitors

**DOI:** 10.1186/1471-2105-12-S14-S4

**Published:** 2011-12-14

**Authors:** Shalini John, Sundarapandian Thangapandian, Mahreen Arooj, Jong Chan Hong, Kwang Dong Kim, Keun Woo Lee

**Affiliations:** 1 Division of Applied Life Science (BK21 Program), Systems and Synthetic Agrobiotech Center (SSAC), Research Institute of Natural Science(RINS), Plant Molecular Biology and Biotechnology Research Center (PMBBRC), Gyeongsang National University (GNU), 501 Jinju-daero, Gazha-dong, Jinju 660-701, Republic of Korea

## Abstract

**Background:**

Renin has become an attractive target in controlling hypertension because of the high specificity towards its only substrate, angiotensinogen. The conversion of angiotensinogen to angiotensin I is the first and rate-limiting step of renin-angiotensin system and thus designing inhibitors to block this step is focused in this study.

**Methods:**

Ligand-based quantitative pharmacophore modeling methodology was used in identifying the important molecular chemical features present in the set of already known active compounds and the missing features from the set of inactive compounds. A training set containing 18 compounds including active and inactive compounds with a substantial degree of diversity was used in developing the pharmacophore models. A test set containing 93 compounds, Fischer randomization, and leave-one-out methods were used in the validation of the pharmacophore model. Database screening was performed using the best pharmacophore model as a 3D structural query. Molecular docking and density functional theory calculations were used to select the hit compounds with strong molecular interactions and favorable electronic features.

**Results:**

The best quantitative pharmacophore model selected was made of one hydrophobic, one hydrogen bond donor, and two hydrogen bond acceptor features with high a correlation value of 0.944. Upon validation using an external test set of 93 compounds, Fischer randomization, and leave-one-out methods, this model was used in database screening to identify chemical compounds containing the identified pharmacophoric features. Molecular docking and density functional theory studies have confirmed that the identified hits possess the essential binding characteristics and electronic properties of potent inhibitors.

**Conclusion:**

A quantitative pharmacophore model of predictive ability was developed with essential molecular features of a potent renin inhibitor. Using this pharmacophore model, two potential inhibitory leads were identified to be used in designing novel and future renin inhibitors as antihypertensive drugs.

## Background

Hypertension is a major factor concerning various cardiovascular diseases such as congestive cardiac failure, stroke, and myocardial infarction and affects up to 30% of the adult population in most countries [1]. Renin is an aspartyl protease and catalytically similar to other enzymes such as pepsin, cathepsin and chymosin etc [2]. Renin cleaves the angiotensinogen to angiotensin-I which is then converted to angiotensin-II by the action of angiotensinogen converting enzyme (ACE). Angiotensin-II is a biologically active vasopressor recognized by its receptors which is one of the cascades of events that leads to the increase in blood pressure. Renin is synthesized as prorenin, a proenzyme, which is transformed into mature renin by the cleavage of 43 amino acids long prosegment from the N-terminal end. This conversion of prorenin to renin occurs in the juxtaglomerular cells of kidney followed by the release of renin into the circulation [3]. Renin blocks the first and rate-limiting step which is the conversion of angiotensinogen to angiotensin-I. Renin is a very specific enzyme towards its only known substrate, angiotensinogen, and this remarkable specificity makes it a very attractive and ideal target to block the renin-angiotensin system (RAS) [4]. Inhibition of renin prevents the formation of both angiotensin-I and II but this is not the case in ACE inhibitors and angiotensin receptor blockers, which increase angiotensin-I or/and II level, respectively. Only renin inhibitors will render the complete RAS quiescent by suppressing the first step of the cascade of events. Thus, inhibition of renin would favor more complete blockade of the system [5]. Potent inhibitors of this enzyme could therefore provide a new alternative way to treat hypertension without inhibiting other biological substances. Aspartyl protease class of enzymes contains two aspartic acid residues that are necessary for the activity. Renin enzyme has a bilobal structure similar to other aspartic proteases and an active site at the interface. The two important aspartate residues Asp32 and Asp215 catalyze the proteolytic function of renin are donated from each lobes of the enzyme [6]. The active site of renin appears as a long, deep cleft that can accommodate seven amino acid units of the substrate, angiotensinogen, and cleaves the peptide bond between Leu10 and Val11 within angiotensinogen to generate angiotensin-I [7]. The approaches followed to develop early renin inhibitors were based on two methodologies. One is to develop similar peptides to prorenin as this segment covers the active site of renin prior to the maturation. The second is based on the N-terminal portion of the substrate, angiotensinogen, for this binds the active site of renin. But these approaches produced only weak inhibitors [8]. The first synthetic renin inhibitor was pepstatin. First-generation renin inhibitors were peptide analogues of the prosegment of renin or substrate analogues of the amino-terminal sequence of angiotensinogen containing the renin cleavage site [9].Crystal structure analyses of renin-inhibitor complexes and computational molecular modeling were later used to design selective nonpeptide renin inhibitors that lacked the extended peptide-like backbone of previous inhibitor sand had improved pharmacokinetic properties [10]. Aliskiren is the first of these new nonpeptide inhibitors to be approved by the FDA for the treatment of hypertension but its synthesis include many steps. This invites much simpler compounds to be designed as potent renin inhibitors [11]. Aliskiren belongs to the third generation of renin inhibitors where the large (high molecular weight) first and second generation inhibitors could not be exploited as drugs despite of their potency *in vitro *[12]. To date, only few compounds were successfully developed with potent renin inhibition profiles, high efficacy, and safety. Thus designing inhibitors of high potential for renin inhibition is the most effective way to block the RAS completely. This study was focused to identify novel scaffolds with the potential to turn as the new category of renin inhibitors.

A high-correlation quantitative pharmacophore model was generated, in this study, using the observed structure-activity relationship of known renin inhibitors. We have successfully applied pharmacophore modeling, database screening, molecular docking, and density functional theory (DFT) calculation methodologies in identifying lead candidates to be employed in potent renin inhibitor design and thereby new category of anti-hypertensive agents.

## Methods

### Selection of training set compounds

**Figure 1:**
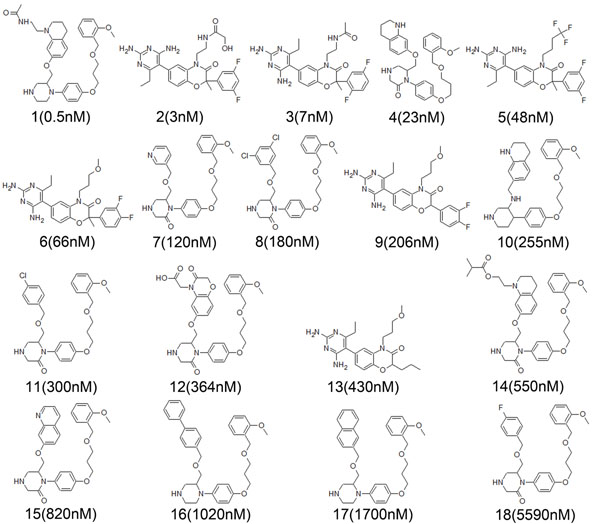
**Structure of the training set compounds.** 2D Chemical structures of the 18 training set compounds together with their experimental IC_50_ values.

Three dimensional (3D) QSAR strategy is one of the ligand-based pharmacophore modeling approaches. This strategy differs from the common feature pharmacophore approach in various points such as limitations of number of training set compounds and necessity of experimental activity values predicted using similar bioassay conditions etc [13]. More than 300 chemical compounds were retrieved from various literature resources [14-19] and 111 compounds evaluated with the same bio-assay protocol were selected to be used as primary data set in 3D QSAR pharmacophore modeling study. To ensure the statistical relevance, a training set containing 18 diverse compounds with the experimental activity values (IC_50_) ranging from 0.5 nM to 5590 nM were selected from 111 dataset compounds and used as training set (Figure 1) and the remaining 93 compounds were used as test set compounds to be utilized in pharmacophore validation.

### Compounds preparation and conformation generation

The two-dimensional (2D) chemical structures of all the compounds in the data set were sketched using ChemSketch, version 12 (ACD Inc., Toronto, Canada) and subsequently converted to 3D structures in Accelrys Discovery Studio 2.5 (DS). These 3D compounds were further checked for the added hydrogens and minimized using smart minimizer that performs 1000 steps of steepest descent followed by conjugate gradient algorithms with a convergence gradient of 0.001 kcal mol^-1^. After energy minimization, multiple acceptable conformers were generated for every training set compound within DS *Diverse Conformation Generation* module using the Poling algorithm. This step was necessary to produce a good set of representative conformations of different conformation space accessible to a molecule within a given energy range. A maximum of 255 conformations were generated for each compound within an energy range of 20 kcal mol^-1^ above the global energy minimum [20-22].

### Generation of pharmacophore models

Among the two types of ligand-based pharmacophore modeling methodologies, common feature pharmacophore modeling utilizes the common chemical features present only in the most active compounds whereas the 3D QSAR pharmacophore methodology uses the chemical features of most active and inactive compounds along with their biological activity. In this study, we have employed 3D QSAR-based pharmacophore methodology to generate pharmacophore models that can be used to estimate the activity of newly designed compounds. *Feature mapping* protocol as available in DS was used to identify the features that are present in the training set compounds. Uncertainty value was set to 2 and the minimum inter-feature distance was set to 2Å from the default value of 2.97 Å. As identified by the feature mapping protocol, hydrogen bond acceptor (HBA), hydrogen bond donor (HBD), hydrophobic aliphatic (HY-AL), hydrophobic aromatic (HY-AR) and ring aromatic (RA) features were used with other default values to generate ten pharmacophore models using *3D QSAR pharmacophore generation* of DS. Each feature of the resulting models occupies a certain weight that is proportional to its relative contribution to biological activity. HypoGen therefore constructs pharmacophore models correlating best with biological activities and consisting of as few features as possible. The HypoGen pharmacophore model generation process is performed in three steps such as the constructive phase, the subtractive phase and the optimization phase [23,24]. Hypotheses that are common to the most active set of compounds are identified during the constructive phase. HypoGen calculates all possible pharmacophore configurations using all combinations of pharmacophore features for each of the conformations of the two most active compounds. Additionally, the hypotheses must fit a minimum subset of features of the remaining most active compounds in order to be considered. A large database of pharmacophore configurations is generated at the end of the constructive phase. In the subtractive phase, all pharmacophore configurations that are also present in the least active set of molecules are removed. All compounds whose activity is by default 3.5 orders of magnitude less than that of the most active compound are considered to represent the least active molecules. The value 3.5 is adjustable depending on the activity range of the training set. During the optimization phase, the hypothesis score is improved. Hypotheses are scored based on errors in activity estimates from regression and complexity. The optimization involves a variation of features and/or locations to optimize activity prediction via a simulated annealing approach. When the optimization process no longer improves the score, HypoGen stops and reports the top scoring 10 unique pharmacophores. The generated pharmacophore models were evaluated for their reliability based on the cost parameters. The overall costs of a model consist of three cost components, namely, the weight cost, the error cost, and the configuration cost. The weight component is a value that increases in a Gaussian form as this function weights in a model deviate from the ideal value of two. The error cost represents the difference between estimated and measured activities of the training set. The configuration cost quantifies the entropy of the hypothesis space.

In addition, the following three cost values are calculated during the generation of pharmacophore models: the fixed cost, the total cost, and the null cost. The fixed cost is the lowest possible cost representing a hypothetical simplest model that fits all data perfectly. Fixed costs are calculated by adding the minimum achievable error and weight cost and the constant configuration cost. Another cost parameter, the null cost, represents the maximum cost of a pharmacophore with no features and estimates activity to be the average of activity data of training set molecules. The null cost value is equal to the maximum occurring error cost. For every pharmacophore generation ten total cost values and each of fixed cost and null cost values are calculated by the pharmacophore generation protocol in the unit of bits. For a meaningful pharmacophore model, the fixed cost should be lower and the null cost should be higher and the total cost value should be closer to the fixed cost and away from the null cost value [25,26]. HypoGen further estimates the activity of each training set compound using regression parameters. The parameters were computed by regression analysis using the relationship of geometric fit value versus the negative logarithm of activity. The better the geometric fit the greater the activity prediction of the compound. Along with these cost values, other statistical values such as correlation coefficient and root mean square deviation (RMSD) were calculated. The best pharmacophore model was selected based on the large cost difference, high correlation coefficient and lower RMSD.

### Pharmacophore validation

The main purpose to validate the generated pharmacophore models is to investigate their ability to estimate the activity of new compounds identified through database screening or designed *de novo*. The selected pharmacophore model was validated using three methods based on the derived cost components, ability in test set prediction, Fischer randomization test results, and leave-one-out method. A larger difference between the fixed and null costs than that between the fixed and total costs signifies the quality of a pharmacophore model. All of these cost values are reported in bits and a difference of 40-60 bits between the total and null costs suggests a 75-90% chance of representing a true correlation in the data [27,28]. Ninety three diverse compounds were used as the test set to validate the pharmacophore model. Fischer randomization is another approach for pharmacophore model validation. The 95% confidence level was selected in this validation study and 19 random spreadsheets were constructed. This validation method checks the correlation between the chemical structures and biological activity. This method generates pharmacophore models using the same parameters as those used to develop the original pharmacophore model by randomizing the activity data of the training set compounds. Finally the cross validation of the model was performed by using the leave-one-out methodology. In this method, 18 pharmacophore models were generated with the same parameters used for generating original pharmacophore model but leaving one compound at a time from 18 training set compounds to ensure the influence of every single training set compound in the generation of selected pharmacophore model [29,30].

### Database screening and drug-like prediction

The best pharmacophore model validated using different methods was used as a 3D query in database screening to retrieve chemical compounds that fit all the pharmacophoric features. A chemical compound must fit all the features to be picked as hits. *Search 3D Database* protocol with *Best/Flexible* search option was employed in database screening. Three chemical databases of diverse chemical compounds were screened for novel chemical scaffolds to be used in potent renin inhibitor design. The identified database hits were screened using various filters based on estimated activity, Lipinski’s rule of five [31], and ADMET properties [32-35].

### Molecular docking

Compounds satisfying all the filters were subjected to molecular docking studies. The GOLD (Genetic Optimization for Ligand Docking) program from Cambridge Crystallographic Data Centre, UK uses a genetic algorithm to dock the small molecules into the protein active site was used in molecular docking [36]. GOLD allows for a full range of flexibility for the ligands and partial flexibility of the protein. Protein coordinates from the crystal structure complex of renin with aliskiren (PDB ID: 2V0Z), one of the most active inhibitors, determined at a resolution of 2.20 Å were used to define the active site. The active site was defined with a 10 Å radius around the bound inhibitor. All the water molecules except two catalytically important 184 and 250 were removed from the protein and hydrogens were added. The ten top-scoring conformations of every ligand were saved at the end of the calculation. Early termination option was used to skip the genetic optimization calculation when any five conformations of a particular compound were predicted within an RMSD value of 1.5 Å. The GOLD fitness score is calculated from the contributions of hydrogen bond and van der Waals interactions between the protein and ligand, intramolecular hydrogen bonds and strains of the ligand [37,38]. Protein-ligand interactions were analyzed using DS and Molegro Virtual Docker [39] programs. The novelty of the final hits was confirmed using *SciFinder *[40] and *PubChem *[41] structure search tools.

### Density functional theory (DFT) calculations

The final hits along with some most and least active compounds were used as input and all DFT calculations were carried out using Gaussian version 3.0 program. The geometry optimization of a set of compounds was carried out using the Becke3 Lee-Yang-Parr correlation functional (B3LYP), at the 6-31G* level [42-45]. The orbital energies of frontier orbitals, namely, highest occupied molecular orbital (HOMO) and lowest unoccupied molecular orbital (LUMO) were calculated for a set of compounds. The calculation was performed to evaluate the electronic properties of final hits to be compared with the compounds in the training set [46].

## Results and discussion

### Pharmacophore generation

**Figure 2:**
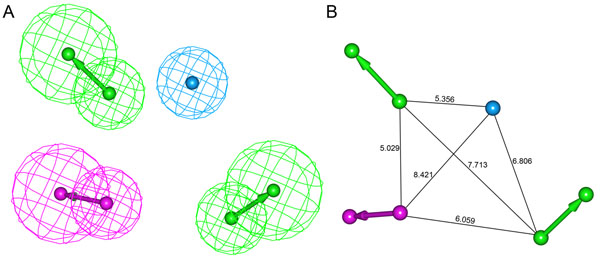
**The best *HypoGen *pharmacophoremodel, Hypo1.** (A) Chemical features present in Hypo 1 (B) 3D spatial arrangement and the distance constraints between the chemical features. Green color represents HBA, magenta color represents HBD and cyan color represents HY-AL features.

A set of ten pharmacophore models was generated using a training set containing 18 compounds by selecting HBA, HBD, HY-AL, HY-AR and RA features as suggested by *Feature Mapping* protocol. All the generated pharmacophore models composed of either HBA or HBD or both with HY-AL or HY-AR features. The total cost values of ten pharmacophore models ranged from 81.50 to 99.54. The cost difference between the total cost and null cost must be greater and it should be smaller between total cost and fixed cost values for a significant pharmacophore model. In our study, the pharmacophore generation run calculated a fixed cost value of 70.08 and the null cost value of 148.56. Among the total cost values of generated ten pharmacophore models, first model (Hypo1) has scored the value closer to the fixed cost value when compared to other models. The cost difference between the null cost and total cost value of the first pharmacophore model is 67.06 (Table 1). The cost difference value between 40 and 60 implies that the pharmacophore model correlates the experimental and estimated activity values more than 90%. In this study, the cost difference value of Hypo1 signifies that it can correlate the experimental and estimated activity values of the training set compounds more than 90%. Hypo1 was made of four pharmacophoric features consisting two HBA, one HBD and one HY-AL features (Figure 2).

**Table 1:** Statistical results of the top 10 pharmacophore hypotheses generated by *HypoGen* algorithm.

Hypothesis	Total cost	Cost difference	RMSD	Correlation	Features
Hypo1	81.50	67.06	1.080	0.944	HBA, HBA, HBD, HY-AL
Hypo2	88.05	60.51	1.247	0.928	HBD, HY-AL, HY-AL, HY-AR
Hypo3	90.28	58.28	1.388	0.908	HBA, HBA, HY-AL, HY-AR
Hypo4	92.63	55.93	1.565	0.878	HBA, HBD, HY-AR, HY-AR
Hypo5	95.59	52.97	1.596	0.875	HBA, HBA, HY-AL, HY-AR
Hypo6	97.63	50.93	1.737	0.847	HBD, HBD, HY-AL, HY-AL
Hypo7	97.71	50.85	1.720	0.851	HBA, HBD, HY-AL, HY-AR
Hypo8	98.80	49.76	1.662	0.866	HBA, HBD, HY-AL, HY-AR
Hypo9	98.85	49.71	1.631	0.873	HBD, HBD, HY-AL, HY-AR
Hypo10	99.54	49.02	1.779	0.840	HBA, HBA, HBD, HY-AL

Null cost = 148.56; fixed cost = 70.08; configuration cost = 16.7.

^a^Cost difference = null cost – total cost.

^b^HBA, hydrogen-bond acceptor; HBD, hydrogen-bond donor; HY-AL, hydrophobic aliphatic; HY-AR, hydrophobic aromatic.

**Figure 3:**
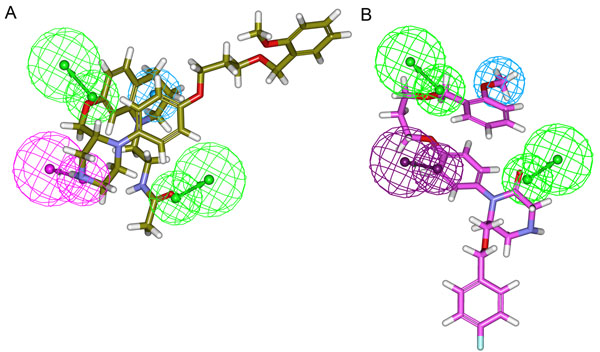
**Pharmacophore Mapping.** (A) Mapping of the most active compound 1 in the training set on the best pharmacophore model Hypo1. (B) Mapping of the least active compound 18 in the training set on the best pharmacophore model Hypo1. In the pharmacophore model green represents HBA, magenta represents HBD and cyan represents HY-AL features.

Further evaluation of the generated pharmacophore models was based on the correlation coefficient. The correlation values of these ten pharmacophore models were greater than 0.840, and the first three pharmacophore models correlated the activity data with high correlation values, i.e., above 0.9. These results indicate the capability of the pharmacophore model to predict the activity of the training set compounds. Hypo1 showed the highest correlation coefficient value of 0.944, highlighting its strong predictive ability. In addition, RMSD values for the top three pharmacophore models were less than 1.5, further supporting the predictive ability of the top pharmacophore models. Among the ten pharmacophore models, Hypo1 was developed with better statistical values, such as higher correlation, large cost difference and lower RMSD (1.080). Hypo1 has predicted the experimental activity values of training set compounds with high correlation. All of the compounds in the training set and test set were categorized into four different groups based on their experimental activity (IC50) values: most active (IC50 ≤ 10nM, ++++), active (10< IC50 ≤ 200nM, +++), moderately active (200< IC50 ≤ 1000 nM, ++), and inactive (IC50 >1000nM, +). The predictive ability of Hypo1 on training set compounds is shown in Table 2. Activity values of 15 out of 18 compounds in the training set were predicted within their experimental activity scale where compound 9, 13, and 14 were overestimated as active. None of the calculated error values that represent the ratio between the experimental and predicted activity values were more than one order of magnitude. All of the three most active compounds in the training set were predicted very closely to their activity values indicating the predictability of Hypo1. The most active compounds in training set mapped all the features of Hypo1 whereas the other compounds miss any one of the pharmacophoric features. The pharmacophore mapping of most and least active compounds are shown in Figure 3. The Hypo1 was selected as best model over Hypo2 which has also shown a high correlation value (0.928) close to that of Hypo1 (0.944) because of the HBA features. These HBA features generated in Hypo1 was similar to the structure-based pharmacophore model developed by our group recently [47]. These important HBA features are not generated in Hypo2. In addition, the energy values of the conformations of the most active compounds in the training set used in model generation were lower in Hypo1 but relatively higher in Hypo2. These analyses have also supported the reliability of Hypo1 along with the high correlation coefficient.

**Table 2:** Experimental and estimated IC_50_ values of the training set compounds based on best pharmacophore hypothesis Hypo1.

Name	IC50 nM		Error^a^	Fit value^b^	Activity scale^c^	
				
	Experimental	Estimated			Experimental	Estimated
1	0.5	0.40	-1.3	9.30	++++	++++
2	3	3.7	1.2	8.33	++++	++++
3	7	7.3	1.0	8.03	++++	++++
4	23	158	6.9	6.69	+++	+++
5	48	130	2.7	6.77	+++	+++
6	66	148	2.2	6.72	+++	+++
7	120	164	1.4	6.67	+++	+++
8	180	151	-1.2	6.71	+++	+++
9	206	144	-1.4	6.72	++	+++
10	255	235	-1.1	6.52	++	++
11	300	414	1.4	6.27	++	++
12	364	417	1.1	6.28	++	++
13	430	126	-3.4	6.80	++	+++
14	550	187	-2.9	6.62	++	+++
15	820	501	-1.6	6.19	++	++
16	1020	1280	1.3	5.78	+	+
17	1700	1088	-1.6	5.85	+	+
18	5590	1838	-3.0	5.63	+	+

^a^Positive value indicates that the estimated IC_50_ is higher than the experimental IC_50_; negative value indicates that the estimated IC_50_ is lower than the experimental IC_50_.

^b^Fit value indicates how well the features in the pharmacophore map the chemical features in the compound.

^c^Activity scale: IC_50_ ≤10nM (Most active, ++++); 10 < IC_50_ ≤ 200nM (Active, +++); 200 < IC_50_ ≤ 1000nM (Moderately active, ++); > 1000nM (Inactive, +).

### Pharmacophore validation

**Figure 4:**
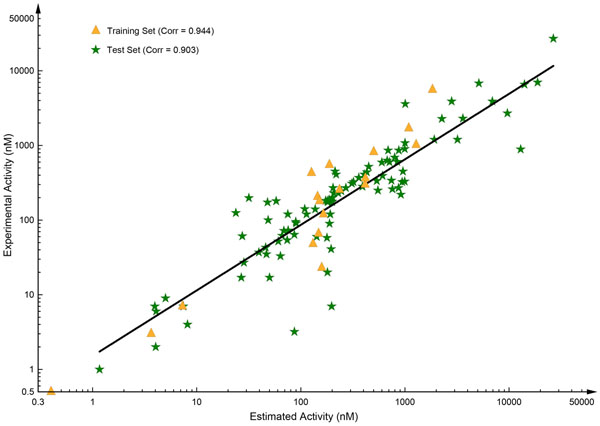
**Correlation plots.** Correlation graph between the experimental activity and the estimated activity for the training set and test set compounds.

The best pharmacophore model, Hypo1, was validated using 93 test set compounds, which are diverse comparing to the training set compounds. The *Ligand Pharmacophore Mapping* protocol with the *Best Flexible Search* option was used to map every test set compound and the estimated activity values were predicted for each compound. The simple regression between the experimental and estimated activity values of the test set compounds showed a correlation coefficient value of 0.903 (Figure 4). Out of 93 test set compounds, five compounds were predicted in a different activity scale with a success rate of 94.62%. Two ‘most active’ compounds were underestimated to ‘active’ scale and one compound from the ‘active’ scale was underestimated in ‘moderately active’ scale. Two ‘moderately active’ compounds were underestimated in ‘inactive’ scale. All compounds from the ‘inactive’ scale were predicted within their activity scale (1 Table A1).

In addition, Hypo1 was further validated using Fischer randomization test to testify that this pharmacophore model is not resulted due to the random correlation. The experimental activities of the training set were scrambled randomly and the resulting training set was used in HypoGen with the parameters chosen for the original pharmacophore generation. A set of 19 random spreadsheets was generated to achieve a 95% confidence level that the best pharmacophore Hypo1 was not generated by chance. None of the randomly generated pharmacophore models during Fischer randomization test has scored better statistical parameters than Hypo1. Though there were five random pharmacophores scored a correlation value above 0.9 none of their RMSD values were lower than Hypo1 (Table 3).

**Table 3:** Results of Fischer’s randomization test.

Validation no.	Total cost	Fixed cost	RMSD	Correlation
Original hypothesis				
Hypo1	81.50	70.08	1.080	0.944
Randomized hypotheses				
Trial1	95.892	70.080	1.661	0.862
Trial2	100.32	70.212	1.708	0.857
Trial3	92.248	65.837	1.531	0.890
Trial4	87.507	67.786	1.421	0.902
Trial5	93.581	68.931	1.653	0.863
Trial6	89.344	71.436	1.299	0.920
Trial7	87.886	66.944	1.464	0.895
Trial8	101.135	70.080	1.633	0.877
Trial9	98.448	68.674	1.764	0.844
Trial10	94.837	65.837	1.649	0.868
Trial11	99.687	71.629	1.663	0.865
Trial12	91.806	69.736	1.562	0.877
Trial13	107.608	70.080	2.021	0.787
Trial14	92.806	69.736	1.470	0.897
Trial15	101.133	69.688	1.866	0.822
Trial16	87.621	71.436	1.340	0.912
Trial17	90.393	70.212	1.357	0.913
Trial18	84.474	65.837	1.377	0.908
Trial19	98.957	70.268	1.722	0.852

The final validation was performed using leave-one-out method, this method is used to verify whether the correlation between the experimental and predicted activities is mainly depend on one particular molecule in the training set. This is done by recomputing the pharmacophore model by excluding one molecule at a time. Consequently, 18 HypoGen calculations were carried out under the same conditions, used in the generation of original pharmacophore model Hypo1, by deriving 18 new training sets, each composed of 17 molecules. The result is positive if none of the correlation coefficients of newly generated pharmacophore models is higher or too lower to that of Hypo1. From our results it was observed that none of the 18 new models generated by this method has shown any meaningful difference compared to Hypo1 (data not shown). This result supports and increases the confident level of Hypo1 that its correlation coefficient does not depend on one particular compound in the training set. Based on these validation results, Hypo1 was used as 3D query in database screening to identify the diverse chemical compounds to be utilized in potent renin inhibitor design.

### Database screening

**Figure 5:**
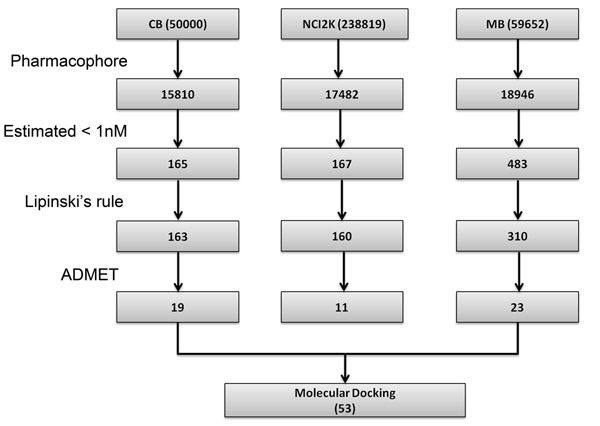
**Database screening.** The flow of procedure used in 3D QSAR pharmacophore modeling.

The best pharmacophore model, Hypo1, was used as a 3D query to search three chemical databases, namely, NCI (260,071 compounds), Maybridge (59,632) and Chembridge (50,000) containing totally 369,703 compounds. *Search 3D Database* protocol with the *Best Search* option as available in DS was employed to search these databases. The hit compounds were, primarily, filtered based on the estimated activity value followed by drug-likeness prediction. Lipinski’s rule of five and ADMET properties were used to select the compounds with favorable drug-like properties. A compound has to obey the following parameters to be predicted as Lipinski-positive: (i) molecular weight less than 500 (ii) number of hydrogen bond donors and acceptors less than 5 and 10, respectively and (iii) octanol / water partition coefficient value less than 5. Fifty-three drug-like compounds along with the training set compounds were subjected to molecular docking study. Database screening and drug-likeness prediction steps are shown in detail in Figure 5.

### Molecular docking

**Figure 6:**
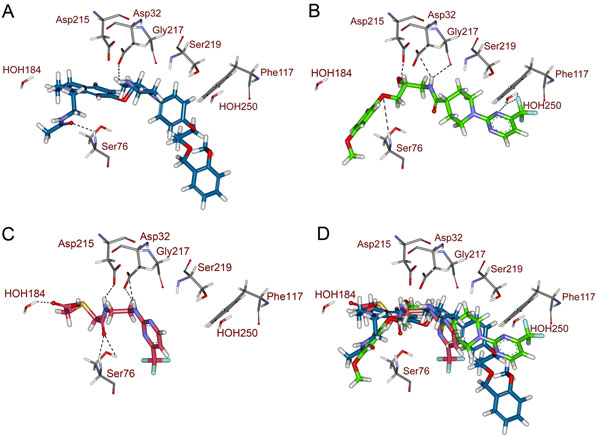
**Molecular docking experiments of 3D QSAR pharmacophore modeling.** Compound 1, the most active compound in the training set is shown in blue color whereas HTS05096 and AW00695 are shown in green and red colors, respectively. Molecular overlay of all the three compounds is shown at lower right figure. Hydrogen bond interactions are shown in black dotted lines.

**Figure 7:**
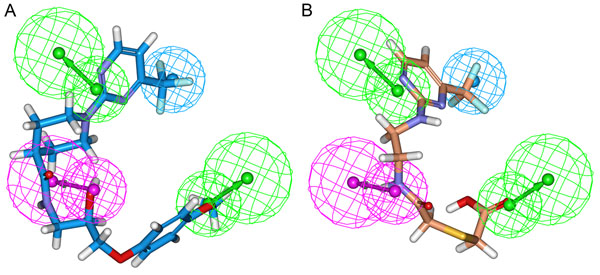
**Pharmacophore mapping of final database hit compounds on the best pharmacophore model Hypo1.** (A) HTS05096 represented in blue color (B) AW00695 represented in orange color. In the pharmacophore model green represents HBA, magenta represents HBD and cyan represents HY-AL features.

**Figure 8:**
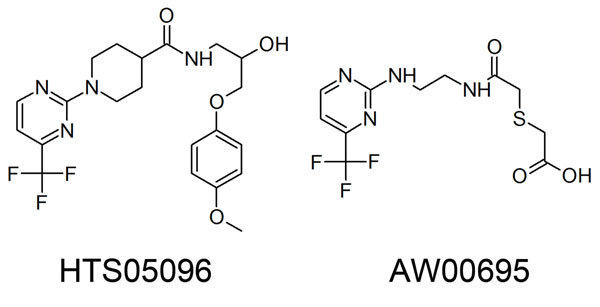
**Chemical structures of hit compounds.** 2D representation of final hits HTS05096 and AW00695.

The drug-like hit compounds along with the training set compounds were docked into the active site. The active site was defined based on the bound inhibitor, aliskiren, in a crystal structure of renin (PDB code 2V0Z). The binding modes, molecular interactions with the active site components and GOLD fitness scores were considered as important components in selecting the best poses of the docked compounds. The active site residues were identified from the protein-ligand interactions observed from the aliskiren-renin complex. Based on the molecular interactions of aliskiren, the new database hits were evaluated. Along with two catalytically important aspartate residues, two active site water molecules were also given importance as aliskiren interacts with them. Compound 1 in the training set has scored a GOLD fitness score of 41.22 and formed hydrogen bond interactions with Asp32 and Gly217. It has also interacted hydrophobically with other active site amino acids (Figure 6A). Twenty-five hit compounds scoring a GOLD fitness score value greater than that of Compound 1 were selected and their binding modes and molecular interactions were analyzed. Finally, two compounds namely HTS05096 and AW00695 from Maybridge database were chosen based on hydrogen bond interactions with two aspartic acid residues and one of two water molecules as well as the other active site residues. HTS05096 has scored the Hypo1estimated activity value of 0.78 along with the GOLD fitness score of 49.38. This compound has formed hydrogen interactions with both the aspartic acid residues and a water molecule in the active site (Figure 6B). It has also formed hydrophobic interactions with Phe117 and other active site residues. The second hit, AW00695, interacted with aspartic acid residues (Asp32 and Asp215), S76 and one of the active site water molecule, HOH184, as well as the hydrophobic interactions with active residues (Figure 6C). The pharmacophore overlay of this hit revealed that the parts overlaid on HBD and HBA features are involved in polar contacts with aspartate and serine residues in the active site whereas the HY-AL part is positioned towards the Phe117 enabling hydrophobic interactions. Figure 6D shows the overlay of compound 1 and two hit compounds in the active site. The pharmacophore mapping and 2D representation of these compounds are shown in Figs. 7 and 8, respectively.

The interaction between the protein and the ligand molecules were observed using DS and Molegro Virtual Docker. The novelty of the two final hit compounds was confirmed using S*ciFinder* search and *PubChem* search.

### Density Functional Theory calculations

**Figure 9:**
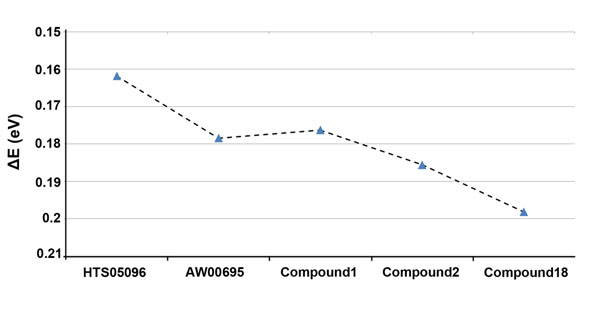
**The plot of energy gaps.** The ΔE (LUMO-HOMO) values of two hit compounds along with the 2 most active and 1 least active compounds of the training set.

The DFT calculations for a set including two final hits, two most active, and a least active compounds were performed in order to study the electronic properties such as HOMO and LUMO. The values of energy gaps (ΔE) were calculated for all the compounds. The energy gaps have been calculated as the difference of the energies between the LUMO and the HOMO. The energy gap values of the 5 compounds ranged from 0.16174 to 0.19811 eV. The energy gap between the LUMO and HOMO elucidates the reactivity of the molecule that is the smaller the gap and the more reactive is the molecule [48,49]. Figure 9 shows the plot of energy gap values calculated for two final hits, two most active and a least active compounds. The Hit1, HTS05096, showed the minimum energy gap value of 0.163 and the least active compound 18 showed the maximum energy gap value of 0.198. Hit 2, AW00695, has shown a value of 0.179 whereas the two most active compounds, namely compound 1 and 2, have shown the energy gap values of 0.178 and 0.186, respectively. This observation revealed that the final hit compounds have shown smaller energy gap values than the most active compounds thereby indicated their high reactivity. The calculated energy gap values have shown good correlation with the biological activity values. From this analysis it was observed that the final hit compounds have displayed the better or similar electronic properties compared to the most active compounds in the training set. These results have also provided the confidence on the quality of the developed pharmacophore model, Hypo1.

## Conclusions

In the present work, a quantitative pharmacophore model, Hypo1, was developed based on the training set compounds with a high diversity in terms of chemical structures and biological activity values. The best pharmacophore model was selected based on various parameters such as cost difference, correlation co-efficient and validation results. Hypo1 was generated with one HY-AL, one HBD and two HBA features with a high correlation coefficient value of 0.944. The validation methods included test set prediction, Fischer randomization, and leave-one-out method. The external test set containing 93 compounds was used in validating the ability of Hypo1 in predicting the activities of compounds that are not included in training set. Hypo1 has predicted this test set with a high correlation value of 0.903. The second validation based on Fischer randomization has proved that Hypo1 was not generated by a chance correlation in the training set. The leave-one-out validation proved that the correlation coefficient of Hypo1 did not depend on one particular compound in the training set. All these validation procedures have shown the strength of the selected model, Hypo1, in predicting the active compounds. After observing the validation results, Hypo1 was used in database screening to identify hits that can be used in potent renin inhibitor design. The identified hit compounds were further filtered based on the binding mode and molecular interactions at the active site of renin. The final hits reported as potential lead compounds have scored high estimated activity, favorable drug-like properties and strong molecular interactions with the catalytic residues at the active site. The DFT calculations were performed to study the electronic properties of the hit compounds and thereby to validate the quality of the pharmacophore model, Hypo1. The final hits, HTS05096 and AW00695, showed the minimum energy gaps which represent the more reactivity of the hit compounds when compare to the most active compounds. This provided the confidence on the inhibitory property of the final hit compounds. Thus, these hits can be utilized in designing future class of novel renin inhibitors.

## Competing interests

The authors declare that they have no competing interests.

## Authors’ contributions

SJ has designed the methodology, performed calculations, analyzed the results, and written the manuscript. ST has involved in analyzing the results and writing the manuscript. MA has performed some of the calculations and corrected the manuscript. JCH and KDK have critically suggested various technical issues during the study and checked the quality of the manuscript. KWL supervised the work and edited the manuscript. All authors have read and approved the manuscript.

## Supplementary Material

Additional file 1 Table A1 Comparison of experimental and estimated IC_50_ values of the test set compounds based on best pharmacophore hypothesis Hypo1. Click here for file
