# Supplementary material for: Development, evaluation and application of 3D QSAR Pharmacophore model in the discovery of potential human renin inhibitors
Source: BMC Bioinformatics. 2011 Dec 14;12(Suppl 14):S4. doi: 10.1186/1471-2105-12-S14-S4 (PMC3287469; doi:10.1186/1471-2105-12-S14-S4)
Supplement: Additional file 1 — Table A1 Comparison of experimental and estimated IC50 values of the test set compounds based on best pharmacophore hypothesis Hypo1. [file 1471-2105-12-S14-S4-S1.docx]

**Table A1 - Comparison of experimental and estimated IC_50_ values of the test set compounds based on best pharmacophore hypothesis Hypo1.**

| Name | IC_50_ nM | | Error^a^ | Activity scale^b^ | | Name | IC_50_ nM | | Error^a^ | Activity scale^b^ | |
| --- | --- | --- | --- | --- | --- | --- | --- | --- | --- | --- | --- |
|  | Expt. | Estd. |  | Expt. | Estd. |  | Expt. | Estd. |  | Expt. | Estd. |
| 19 | 1 | 1.165 | 1.2 | ++++ | ++++ | 66 | 220 | 913.6 | 4.2 | ++ | ++ |
| 20 | 2 | 4.034 | 2.0 | ++++ | ++++ | 67 | 230 | 206.8 | -1.1 | ++ | ++ |
| 21 | 3.2 | 26.72 | 8.3 | ++++ | +++ | 68 | 230 | 228.8 | -1.0 | ++ | ++ |
| 22 | 4 | 8.158 | 2.0 | ++++ | ++++ | 69 | 245 | 845.0 | 3.4 | ++ | ++ |
| 23 | 6 | 4.070 | -1.5 | ++++ | ++++ | 70 | 250 | 550.6 | 2.2 | ++ | ++ |
| 24 | 7 | 7.441 | 1.1 | ++++ | ++++ | 71 | 260 | 757.8 | 2.9 | ++ | ++ |
| 25 | 7 | 67.62 | 9.7 | ++++ | +++ | 72 | 270 | 350.7 | 1.3 | ++ | ++ |
| 26 | 7 | 3.956 | -1.8 | ++++ | ++++ | 73 | 270 | 867.7 | 3.2 | ++ | ++ |
| 27 | 9 | 5.013 | -1.8 | ++++ | ++++ | 74 | 270 | 203.8 | -1.3 | ++ | ++ |
| 28 | 17 | 26.80 | 1.6 | +++ | +++ | 75 | 282 | 390.4 | 1.4 | ++ | ++ |
| 29 | 17 | 50.06 | 3.0 | +++ | +++ | 76 | 310 | 308.9 | -1.0 | ++ | ++ |
| 30 | 20 | 180.2 | 9.0 | +++ | +++ | 77 | 325 | 940.0 | 2.9 | ++ | ++ |
| 31 | 27 | 28.38 | 1.1 | +++ | +++ | 78 | 325 | 320.2 | -1.0 | ++ | ++ |
| 32 | 33 | 63.70 | 1.9 | +++ | +++ | 79 | 330 | 993.6 | 3.0 | ++ | ++ |
| 33 | 35 | 46.74 | 1.3 | +++ | +++ | 80 | 336 | 536.8 | 1.6 | ++ | ++ |
| 34 | 37 | 39.49 | 1.1 | +++ | +++ | 81 | 340 | 740.7 | 2.2 | ++ | ++ |
| 35 | 41 | 195.4 | 4.8 | +++ | +++ | 82 | 370 | 1060.5 | -2.9 | ++ | + |
| 36 | 43 | 45.97 | 1.1 | +++ | +++ | 83 | 393 | 609.2 | 1.6 | ++ | ++ |
| 37 | 52 | 60.76 | 1.2 | +++ | +++ | 84 | 410 | 407.3 | -1.0 | ++ | ++ |
| 38 | 54 | 74.04 | 1.4 | +++ | +++ | 85 | 410 | 218.2 | -1.9 | ++ | ++ |
| 39 | 58 | 178.3 | 3.1 | +++ | +++ | 86 | 440 | 430.1 | -1.0 | ++ | ++ |
| 40 | 60 | 142.1 | 2.4 | +++ | +++ | 87 | 450 | 213.1 | -2.1 | ++ | ++ |
| 41 | 61 | 27.41 | -2.2 | +++ | +++ | 88 | 450 | 957.4 | 2.1 | ++ | ++ |
| 42 | 62 | 66.06 | 1.1 | +++ | +++ | 89 | 520 | 448.1 | -1.2 | ++ | ++ |
| 43 | 64 | 86.64 | 1.4 | +++ | +++ | 90 | 594 | 598.9 | 1.0 | ++ | ++ |
| 44 | 72 | 68.75 | -1.0 | +++ | +++ | 91 | 600 | 710.5 | 1.2 | ++ | ++ |
| 45 | 73 | 150.6 | 2.1 | +++ | +++ | 92 | 606 | 843.1 | 1.4 | ++ | ++ |
| 46 | 90 | 188.7 | 2.1 | +++ | +++ | 93 | 630 | 675.5 | 1.1 | ++ | ++ |
| 47 | 91 | 90.13 | -1.0 | +++ | +++ | 94 | 691 | 795.5 | 1.2 | ++ | ++ |
| 48 | 95 | 89.34 | -1.1 | +++ | +++ | 95 | 860 | 872.3 | 1.0 | ++ | ++ |
| 49 | 100 | 48.52 | -2.1 | +++ | +++ | 96 | 860 | 689.3 | -1.2 | ++ | ++ |
| 50 | 120 | 191.6 | 1.6 | +++ | +++ | 97 | 890 | 8536.2 | 9.6 | ++ | + |
| 51 | 120 | 113.4 | -1.1 | +++ | +++ | 98 | 900 | 995.6 | 1.0 | ++ | ++ |
| 52 | 120 | 74.99 | -1.6 | +++ | +++ | 99 | 1080 | 1002.3 | -1.1 | + | + |
| 53 | 125 | 23.74 | -5.3 | +++ | +++ | 100 | 1200 | 3199.6 | -2.7 | + | + |
| 54 | 140 | 137.3 | -1.0 | +++ | +++ | 101 | 1200 | 1912.8 | 1.6 | + | + |
| 55 | 141 | 108.9 | -1.3 | +++ | +++ | 102 | 1600 | 12007.0 | -7.5 | + | + |
| 56 | 173 | 47.89 | -3.6 | +++ | +++ | 103 | 2280 | 2264.5 | -1.0 | + | + |
| 57 | 175 | 181.7 | 1.1 | +++ | +++ | 104 | 2600 | 3604.3 | 1.4 | + | + |
| 58 | 175 | 199.9 | 1.1 | +++ | +++ | 105 | 2700 | 9645.5 | 3.6 | + | + |
| 59 | 178 | 199.1 | -1.1 | +++ | +++ | 106 | 3900 | 6906.8 | 1.8 | + | + |
| 60 | 180 | 179.8 | -1.0 | +++ | +++ | 107 | 3900 | 2807.6 | -1.4 | + | + |
| 61 | 180 | 57.98 | -3.1 | +++ | +++ | 108 | 6560 | 14065.8 | 2.1 | + | + |
| 62 | 182 | 173.2 | -1.1 | +++ | +++ | 109 | 6800 | 5131.7 | -1.3 | + | + |
| 63 | 188 | 187.2 | -1.0 | +++ | +++ | 110 | 7000 | 18789.3 | 2.7 | + | + |
| 64 | 198 | 31.71 | -6.2 | +++ | +++ | 111 | 27000 | 26629.3 | -1.0 | + | + |
| 65 | 200 | 202.4 | 1.0 | +++ | ++ |  |  |  |  |  |  |

^a^Positive value indicates that the estimated IC_50_ is higher than the experimental IC_50_; negative value indicates that the estimated IC_50_ is lower than the experimental IC_50_.

^b^Activity scale: IC_50_ ≤ 10nM (Most active, ++++); 10 < IC_50_ ≤ 200nM (Active, +++); 200 < IC_50_ ≤ 1000nM (Moderately active, ++); > 1000nM (Inactive, +).
